# Association of Attending a High-Performing High School With Substance Use Disorder Rate and Health Outcomes in Young Adults

**DOI:** 10.1001/jamanetworkopen.2022.35083

**Published:** 2022-10-06

**Authors:** Mitchell D. Wong, Benjamin P. L. Meza, Kulwant K. Dosanjh, Nicholas J. Jackson, Teresa E. Seeman, Natalia Orendain, Rebecca N. Dudovitz

**Affiliations:** 1 General Internal Medicine and Health Services Research, David Geffen School of Medicine at UCLA (University of California, Los Angeles); 2 Division of Geriatrics, David Geffen School of Medicine at UCLA; 3 Department of Pediatrics and Children’s Discovery & Innovation Institute, David Geffen School of Medicine at UCLA and UCLA Mattel Children’s Hospital, Los Angeles

## Abstract

**Importance:**

Interventions directly targeting social factors, such as education, may have the potential to greatly improve health.

**Objective:**

To examine the association of attending a high-performing public charter high school with rates of substance use disorder and physical and mental health.

**Design, Setting, and Participants:**

This cohort study used the random school admissions lottery system of high-performing public charter high schools in low-income neighborhoods of Los Angeles, California, to examine the health outcomes of students who applied to at least 1 of 5 of these high schools. Participants attended 147 different high schools and were randomly selected from those who won the admissions lottery (intervention group) and those who were placed on a waiting list (control group). Participants were surveyed at the end of grade 8 through transition into grade 9 and then from grade 10 through 3 years after high school completion (at age 21 years). Surveys were conducted from March 2013 through November 2021.

**Intervention:**

Attendance at a high-performing public charter high school.

**Main Outcomes and Measures:**

Self-reported alcohol use disorder and cannabis misuse, delinquent behaviors, physical and mental health, and body mass index.

**Results:**

Of the 1270 participants at baseline (mean [SD] age, 14.2 [0.47] years; 668 female individuals [52.6%]). The control group included 576 individuals (45.4%), and 694 individuals (54.6%) were in the intervention group. Both groups were similar in almost all characteristics at baseline, and the median (IQR) follow-up was 6.4 (6.0-6.7) years. Participants attending a high-performing public charter high school had a 53.33% lower rate of hazardous or dependent alcohol use disorder compared with those in the control group (5.43% vs 11.64%; difference, −6.21% [95% CI, −11.87% to −0.55%]; *P* = .03). Among male participants, the intervention group had a 42.05% lower rate of self-reported fair or poor physical health (13.33% vs 23.01%; difference, −9.67% [95% CI, −18.30% to −1.05%]; *P* = .03) and a 32.94% lower rate of obesity or overweight (29.28% vs 43.67%; difference, −14.38% [95% CI, −25.74% to −3.02%]; *P* = .02) compared with the control group. Among female participants, attending a high-performing public charter high school was associated with worse physical health outcomes (30.29% vs 13.47% reporting fair or poor health; difference, 16.82% [95% CI, 0.36% to 33.28%]; *P* = .045) and higher rates of overweight or obesity (52.20% vs 32.91%; difference, 19.30% [95% CI, 3.37% to 35.22%]; *P* = .02) at age 21 years. Few differences in mental health outcomes were observed. Adjusting for educational outcomes did not significantly change these findings.

**Conclusions and Relevance:**

Results of this study suggest that attending a high-performing public charter high school was associated with lower rates of substance use disorder independent of academic achievement. Physical health and obesity outcomes were also better but only for young men; the intervention group had worse physical health outcomes among young women for unclear reasons. Schools are a potent social determinant of health and an important target for future health interventions.

## Key Points

**Question:**

Is attendance at a high-performing public charter high school associated with improved health behaviors and health outcomes in young adulthood?

**Findings:**

In this cohort study of 1270 youths, those who attended a high-performing public charter high school reported substantially lower rates of substance use disorder and delinquent behaviors through age 21 years. Attendance at such a school was also associated with substantially better physical health and lower obesity or overweight rates among male participants but substantially worse physical health outcomes among female participants.

**Meaning:**

Findings of this study suggest that improving schools may be an effective and scalable strategy for improving future health outcomes.

## Teaser

This cohort study evaluates the association of attending a high-performing public charter high school with self-reported alcohol and cannabis misuse and physical and mental health in young men and women.

## Introduction

UK professor of epidemiology Michael Marmot once said, “Every minister is a health minister, and every sector is a health sector. If we put fairness at the heart of all policies, health would improve.”^1^ Numerous observational studies suggest that poverty and related social factors are associated with health and well-being from conception to death.^2^ Yet, effective health interventions targeting social factors remain elusive and understudied.

Some small studies have shown an association between early childhood education and an improved range of health outcomes.^3,4,5,6^ The Moving to Opportunities Study found that moving to more affluent neighborhoods improved education and income outcomes for younger children^7^ and had some benefits for girl’s behavioral health.^8^ However, moving led to worse economic outcomes for older children, greater anxiety and substance use for boys, and fewer differences in physical health for either boys or girls.^7,8,9,10,11^

Although intervening on social determinants of health presents substantial challenges, society already invests heavily in education. Public education has also markedly changed during the past 2 decades partly owing to policies, such as the No Child Left Behind Act and Every Student Succeeds Act. Yet, few studies have used more rigorous study designs to examine the association between improved secondary education and health.

In a natural experiment titled the Reducing Inequities Through Social and Educational Change Follow-up (RISE-Up) Study, the admissions lottery system of public charter high schools was used to identify comparable groups of adolescents who were randomized into high- or lower-performing schools.^12^ Outcomes through grade 11 revealed that attending a high-performing high school was associated with a small but significant reduction in cannabis and alcohol misuse and lower risk of having substance-using friends.^12^ In the present study, we examined the longer-term results of the RISE-Up Study through age 21 years. We aimed to examine the association of attending a high-performing public charter high school with rates of substance use disorder and physical and mental health. We hypothesized that exposure to high-performing high schools is associated with lower risk of alcohol use disorder (AUD) and cannabis misuse (primary hypothesis), lower rates of other delinquent behaviors, and better mental and physical health outcomes.

## Methods

### Study Design and Population

This prospective cohort study used the random admission lottery system of high-performing public charter high schools in low-income communities of Los Angeles, California, to identify comparable groups of students randomized to high- and lower-performing public schools. Because the random lottery was not conducted by the investigators, this study is considered a natural experiment rather than a true experiment. We recruited and surveyed young individuals who applied to 1 or more of these high-performing public charter schools. Parental and student consent was obtained from all participants. The University of California, Los Angeles Institutional Review Board approved this study. We followed the Strengthening the Reporting of Observational Studies in Epidemiology (STROBE) reporting guideline.

In fall (October) 2012, we identified from 522 public high schools in Los Angeles County all of the public charter high schools serving low-income communities (n = 91) and those that performed in the top tercile of public schools (n = 30) according to the Academic Performance Index (which is estimated from standardized test scores and rates of attendance and graduation).^13^ Of these 30 schools, we recruited 5 schools with at least 50 more applicants than available spots. Applications were open to all students and required only contact information.

We observed the random admissions lottery system of each of the 5 schools (in spring 2013) and selected students who won the lottery and those who were placed on a waiting list. Unable to recruit enough students in March to November 2013, we repeated recruitment in March to November 2014 using the same procedures. Students who were admitted on the basis of sibling preference and those who moved out of Los Angeles County were excluded from the study.

### Data Collection, Measures, and Covariates

From March 2013 through November 2021, after obtaining parental and student consent, we surveyed participants at the end of grade 8 through the beginning of grade 9 (baseline) and then annually from grade 10 through approximately age 21 years. The fourth (December 2018-April 2020) and the fifth (February 2020-June 2021) follow-up surveys were conducted by telephone or in person. We collected transcripts from the participating schools, standardized test data from the California Department of Education, and college matriculation data from the National Student Clearinghouse.^14^

We used the Alcohol Use Disorders Identification Test (AUDIT) to identify less risky (score <8) vs hazardous or dependent (score ≥8) AUD.^15^ Cannabis misuse was assessed using an 8-item measure adapted from the Alcohol Misuse Scale.^16^ We also assessed previous year engagement in 9 delinquent behaviors (none vs any): graffiti, damaging property, shoplifting or stealing, driving a car without the owner’s permission, burglary, armed robbery, selling illicit drugs, gang participation, and participation in a gang fight.^17^ Self-reported height and weight were used to calculate body mass index (BMI; calculated as weight in kilograms divided by height in meters squared) and BMI percentile.^18,19^ Participants reported their general physical and mental health (fair or poor vs good, very good, or excellent), depression (assessed with the Center of Epidemiological Studies-Depression-10 scale, with score of ≥10 indicating high risk for clinical depression),^20,21^ hopelessness,^22^ self-efficacy,^23^ and generalized anxiety (assessed with the General Anxiety Disorder-7 scale, with a score of ≥10 indicating moderate to severe anxiety).^24^

Participants self-reported baseline demographic characteristics (sex, race and ethnicity [selected from the following categories: American Indian or Native American; Asian or Pacific Islander; Black or African-American; Hispanic; White; or other, including Amerindian, Dutch-Indonesian, French-Creole, Lebanese, and Middle-Eastern], native language, and US birth), parental employment and birthplace, parenting style,^25^ and family structure (1-parent, 2-parent, or other). We collected data on demographic characteristics to be used as covariates in our analyses because they are often associated with differences in health outcomes. Because we recognized that applying to more schools would improve the chances of admission, we adjusted for the combination of charter schools to which a student applied (risk set).^26,27^

### Statistical Analysis

We conducted intent-to-treat (ITT) analyses using mixed-effects models to estimate the association of winning the admissions lottery with health outcomes. To adjust for the hierarchical data structure of students nested within schools, the school was included as a random effect. Previous studies of charter school lotteries estimated the outcome of attending a high-performing school, referred to as the treatment on the treated (TOT), using 2-stage least-squares analysis.^27,28,29^ Using similar methods, admissions lottery assignment served as an instrumental variable because it is random, conditional on the risk set, and thus exogenous to the outcomes. We also conducted longitudinal instrumental variable TOT analyses to examine repeated outcomes measures at all 5 follow-up survey waves, adjusting for clustering of multiple observations per participant.

All models controlled for student demographic characteristics, grade 8 grade point average (GPA), risk set, and parental and family characteristics. We conducted additional analyses adjusted for high school GPA, high school completion, grade 11 standardized test scores, and 4-year college matriculation. We tested for interaction between exposure to a high-performing public charter high school and sex using bootstrapping to estimate 95% CIs. We imputed missing data using multiple imputation with chained equations.

A 2-sided *P* = .05 was used to indicate statistical significance. Stata, version 17.0 (StataCorp LLC), was used for all analyses.

## Results

At baseline, we recruited 1270 students, of whom 576 (45.4%) had been placed on a waiting list (control group) and 694 (54.6%) won the admissions lottery (intervention group). As reported previously,^12^ the control and intervention groups were similar in race and ethnicity, sex, US birth, native English language, parenting style, and family structure. Of the 1270 participants at baseline, 668 were female (52.6%) and 602 were male (47.4%) individuals with a mean (SD) age of 14.2 (0.47) years.

Of the 961 participants at the fifth follow-up survey, 528 were female (54.9%) and 433 were male (45.1%) individuals with a mean (SD) age of 20.8 (0.44) years. Three hundred seventy-one of 420 participants (88.3%) in the control group and 498 of 541 (92.1%) in the intervention group were Latinx individuals, 22 (5.2%) and 19 (3.5%) were non-Latinx Black or African American individuals, and 27 (6.4%) and 24 (4.4%) were non-Latinx White individuals or other (including Amerindian, Dutch-Indonesian, French-Creole, Lebanese, and Middle-Eastern). Most participants in both groups were born in the US (370 [88.1%] in the control group and 468 [86.5%] in the intervention group), and 161 participants (38.3%) in the control group and 218 (40.3%) in the intervention group were native English speakers. Participants were followed up for a median of 6.4 years.

By the fifth follow-up survey (age 21 years), 420 participants (72.9%) in the control group and 541 (78.0%) in the intervention group remained in the study (Table 1; Figure). Those lost to follow-up were more likely to have characteristics that were associated with higher risk of poor outcomes, such as 1-parent family structure, lower grade 8 GPA, and worse standardized test scores (Table 1). However, of the participants who remained through the fifth follow-up survey, the baseline characteristics were similar, with 1 exception: the intervention group was slightly more likely than the control group to have Latinx ethnicity (92.1% [498 of 541] vs 88.3% [371 of 420]; *P* = .05).

**Table 1.** Baseline Demographic Characteristics of the Intervention and Control Groups

Characteristic	Control group			Intervention group			*P* value for retained intervention vs control participants
	Participants, No. (%)		*P* value	Participants, No. (%)		*P* value	
	Retained through the age 21 y survey	Lost to follow-up		Retained through the age 21 y survey	Lost to follow-up		
No. (%)	420 (72.9)	156 (27.1)		541 (78.0)	153 (22.0)		
Female sex	232 (55.2)	67 (42.9)	.009	296 (54.7)	73 (47.7)	.13	.87
Male sex	188 (44.8)	89 (57.1)		245 (45.3)	80 (52.3)		
Race^a^							
American Indian or Native American	3 (0.7)	3 (1.9)	.006	4 (0.7)	4 (2.6)	.003	.25
Asian or Pacific Islander	5 (1.2)	2 (1.3)		13 (2.4)	2 (1.3)		
Black or African American	22 (5.2)	17 (10.9)		24 (4.4)	18 (11.8)		
White	380 (90.5)	133 (85.3)		495 (91.5)	128 (83.7)		
Other or multiracial^b^	10 (2.4)	1 (0.6)		5 (0.9)	1 (0.7)		
Ethnicity^a^							
Latinx	371 (88.3)	129 (82.7)	.08	498 (92.1)	136 (88.9)	.22	.05
Non-Latinx	49 (11.7)	27 (17.3)		43 (7.9)	17 (11.1)		
US-born	370 (88.1)	135 (86.5)	.61	468 (86.5)	140 (91.5)	.10	.46
Native English speaker	161 (38.3)	72 (46.2)	.09	218 (40.3)	68 (44.4)	.36	.54
US-born parent							
No	313 (74.5)	108 (69.2)	.35	408 (75.4)	102 (66.7)	.004	.51
Yes	106 (25.2)	48 (30.8)		133 (24.6)	49 (32.0)		
Do not know	1 (0.2)	0		0	2 (1.3)		
Full-time employed parent							
No	57 (13.6)	25 (16.0)	.63	60 (11.1)	20 (13.1)	.50	.50
Yes	362 (86.2)	131 (84.0)		480 (88.7)	132 (86.3)		
Do not know	1 (0.2)	0		1 (0.2)	1 (0.7)		
Parenting style							
Average	207 (49.3)	73 (46.8)	.87	284 (52.5)	70 (45.8)	.06	.73
Neglectful	86 (20.5)	35 (22.4)		99 (18.3)	44 (28.8)		
Indulgent	41 (9.8)	19 (12.2)		43 (7.9)	14 (9.2)		
Authoritarian	38 (9.0)	12 (7.7)		51 (9.4)	12 (7.8)		
Authoritative	48 (11.4)	17 (10.9)		64 (11.8)	13 (8.5)		
Family structure							
Other	6 (1.4)	6 (3.8)	<.001	12 (2.2)	3 (2.0)	.43	.48
1-parent	65 (15.5)	42 (26.9)		73 (13.5)	27 (17.6)		
2-parent	349 (83.1)	108 (69.2)		456 (84.3)	123 (80.4)		
Grade 8 GPA							
<2.0	41 (9.8)	37 (23.7)	<.001	51 (9.4)	24 (15.7)	<.001	.51
2.0-2.5	80 (19.0)	39 (25.0)		86 (15.9)	42 (27.5)		
2.6-3.0	103 (24.5)	38 (24.4)		119 (22.0)	41 (26.8)		
3.1-3.5	91 (21.7)	30 (19.2)		141 (26.1)	27 (17.6)		
3.6-4.0	95 (22.6)	7 (4.5)		132 (24.4)	18 (11.8)		
Unknown or missing	10 (2.4)	5 (3.2)		12 (2.2)	1 (0.7)		
Standardized test scores							
Grade 8 math (%)							
<Basic level	86 (21.8)	50 (34.0)	.005	115 (22.2)	48 (33.3)	.004	.99
Basic level	97 (24.6)	45 (30.6)		126 (24.3)	40 (27.8)		
≥Proficient level	212 (53.7)	52 (35.4)		277 (53.5)	56 (38.9)		
Grade 8 English (%)							
<Basic level	58 (14.7)	39 (26.5)	<.001	72 (13.9)	26 (18.1)	.01	.18
Basic level	122 (30.9)	59 (40.1)		134 (25.9)	51 (35.4)		
≥Proficient level	215 (54.4)	49 (33.3)		312 (60.2)	67 (46.5)		

Abbreviation: GPA, grade point average.

^a^ Race and ethnicity were self-reported.

^b^ Other was self-identified and included the following responses: Amerindian, Dutch-Indonesian, French-Creole, Lebanese, and Middle-Eastern.

**Figure.**
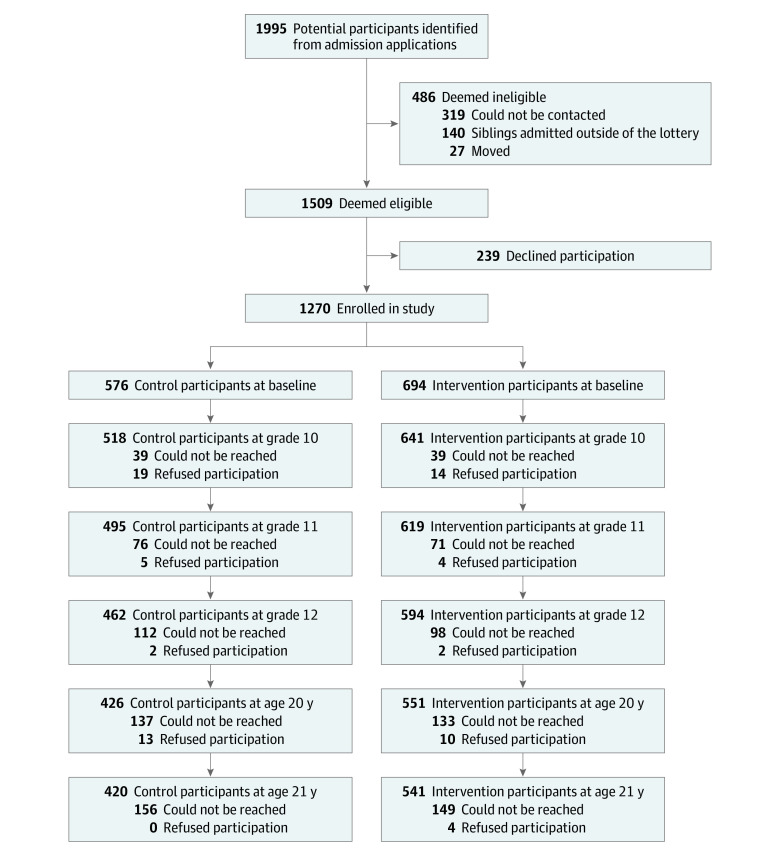
Participant Recruitment and Retention

Of the 694 students in the intervention group, 613 (88.3%) attended a high school in the top tercile of the Academic Performance Index, and 81 (11.7%) attended a lower-performing high school. Of the 576 students in the control group, 357 (62.0%) attended a lower-performing school and 219 (38.0%) attended a high-performing school (*P* < .001).

Overall, adjusted rates of risky behaviors were lower in the intervention than in the control group (Table 2). Specifically, 5.06% of students in the intervention group and 11.38% students in the control group reported hazardous or dependent AUD at age 20 years (TOT model difference, −6.33% [95% CI, −12.02% to −0.63%]; *P* = .03). This difference was smaller and not statistically significant in the ITT model (−3.27% [95% CI, −6.78% to 0.24%]; *P* = .07). Examining both surveys at ages 20 and 21 years when the AUDIT questionnaire was used attending a high-performing public charter high school was associated with lower rates of hazardous or dependent AUD compared to attending a lower-performing school (5.43% vs 11.64%; difference, -6.21% [95% CI, −11.87% to −0.55%]; *P* = .03), and a relative risk reduction of 53.33%. Cannabis misuse scores were also lower (better) among participants in the intervention group at age 20 years (ITT model difference, −3.31 [95% CI, −5.76 to −0.85]; *P* = .008; TOT model difference, −6.40 [95% CI, −11.16 to −1.63]; *P* = .008). Differences in cannabis misuse were not statistically significant at age 21 years; however, across repeated measures from grade 10 through age 21 years, cannabis misuse scores were significantly lower in those who attended a high-performing public charter high school (TOT model longitudinal differences, −3.51 [95% CI, −6.52 to −0.49]; *P* = .02).

**Table 2.** Comparison of Alcohol and Cannabis Misuse and Delinquent Behaviors at Age 20 and 21 Years in the Intervention and Control Groups^a^

Outcome per model	All participants (n = 961)				Female participants				Male participants				*P* value for sex interaction
	Control group (n = 420)	Intervention group (n = 541)	Difference (95% CI)	*P* value	Control group (n = 232)	Intervention group (n = 296)	Difference (95% CI)	*P* value	Control group (n = 188)	Intervention group (n = 245)	Difference (95% CI)	*P* value	
**Hazardous or dependent AUD, %**													
ITT model at age 20 y	8.91	5.64	−3.27 (−6.78 to 0.24)	.07	9.01	6.00	−3.01 (−7.84 to 1.81)	.22	8.89	5.11	−3.78 (−8.90 to 1.34)	.15	.55
TOT model at age 20 y	11.38	5.06	−6.33 (−12.02 to −0.63)	.03	11.72	5.43	−6.29 (−15.73 to 3.16)	.19	11.16	4.50	−6.65 (−15.36 to 2.05)	.13	.97
ITT model at age 21 y	8.86	6.01	−2.85 (−6.80 to 1.09)	.16	8.95	7.38	−1.56 (−7.14 to 4.01)	.58	9.14	6.86	−2.28 (−7.81 to 3.25)	.42	.40
TOT model at age 21 y	11.56	5.46	−6.11 (−12.97 to 0.76)	.08	12.20	4.75	−7.46 (−16.01 to 1.10)	.09	10.53	6.52	−4.02 (−13.97 to 5.94)	.43	.68
Longitudinal TOT	11.64	5.43	−6.21 (−11.87 to −0.55)	.03	11.85	5.12	−6.73 (−14.62 to 1.17)	.09	11.42	5.89	−5.53 (−13.39 to 2.33)	.17	.85
**Cannabis misuse score**													
ITT model at age 20 y	13.43	10.12	−3.31 (−5.76 to −0.85)	.008	11.35	9.77	−1.59 (−4.85 to 1.67)	.34	15.60	11.17	−4.43 (−8.22 to −0.65)	.02	.49
TOT model at age 20 y	15.93	9.53	−6.40 (−11.16 to −1.63)	.008	13.28	9.00	−4.28 (−11.15 to 2.58)	.22	18.27	10.46	−7.81 (−13.09 to −2.53)	.004	.56
ITT model at age 21 y	11.14	10.58	−0.55 (−3.08 to 1.97)	.67	10.00	9.77	−0.23 (−3.33 to 2.86)	.88	12.19	11.14	−1.05 (−5.12 to 3.02)	.61	.50
TOT model at age 21 y	11.69	10.38	−1.30 (−4.82 to 2.22)	.47	10.20	9.73	−0.47 (−5.76 to 4.83)	.86	13.18	11.29	−1.89 (−6.30 to 2.52)	.40	.75
Longitudinal TOT	9.18	5.68	−3.51 (−6.52 to −0.49)	.02	7.96	5.50	−2.46 (−6.52 to 1.60)	.23	10.19	6.06	−4.13 (−8.39 to 0.14)	.06	.74
**Delinquent behaviors, %**													
ITT model at age 20 y	13.62	8.18	−5.44 (−9.94 to −0.95)	.02	12.44	7.02	−5.42 (−10.85 to 0.00)	.05	13.51	9.93	−3.58 (−10.35 to 3.19)	.30	.41
TOT model at age 20 y	18.29	6.82	−11.47 (−20.25 to −2.68)	.01	17.30	6.00	−11.30 (−21.95 to −0.66)	.04	18.21	8.46	−9.76 (−21.55 to 2.04)	.11	.88
ITT model at age 21 y	8.79	5.60	−3.19 (−6.87 to 0.48)	.09	7.16	4.86	−2.31 (−6.72 to 2.11)	.31	10.58	7.39	−3.18 (−8.93 to 2.57)	.28	.88
TOT model at age 21 y	11.29	5.28	−6.01 (−12.14 to 0.11)	.05	9.12	4.46	−4.67 (−13.51 to 4.17)	.30	12.52	6.91	−5.62 (−14.88 to 3.65)	.23	.91
Longitudinal TOT	11.33	10.74	−0.59 (−5.37 to 4.19)	.81	10.33	9.26	−1.07 (−7.45 to 5.30)	.74	12.06	12.77	0.71 (−6.19 to 7.61)	.84	.98

Abbreviations: AUD, alcohol use disorder; ITT, intent-to-treat; TOT, treatment on the treated.

^a^ The ITT analyses were based on mixed-effects models, with the intervention and control group assignments based on the admissions lottery. The TOT models used instrumental variables analysis, with the admissions lottery assignment as the instrument variable and attendance in a high-performing school (defined as school-level test scores in the top tertile of public high schools in Los Angeles County) as the exposure. Longitudinal models used all outcome measures for up to 5 waves of follow-up surveys, adjusted for clustering at the participant level. All models were adjusted for sex, Latino ethnicity, US birth, native English language speaker, grade 8 grade point average, risk set based on which high schools the student applied to, parental birthplace, 1 or more parent with full-time employment status, parenting style at home, and family structure.

The proportion of participants who were engaged in 1 or more delinquent behaviors was lower in the intervention vs control group at age 20 years (ITT model difference, −5.44% [95% CI, −9.94% to −0.95%]; *P* = .02; TOT model difference, −11.47% [95% CI, −20.25% to −2.68%]; *P* = .01) and at age 21 years (ITT model difference, −3.19% [95% CI, −6.87% to 0.48%]; *P* = .09; TOT model difference, −6.01% [95% CI, −12.14% to 0.11%]; *P* = .05). Despite this finding, the proportion of those who engaged in delinquent behaviors was similar between the 2 groups across all follow-up survey waves. The estimated treatment outcome of these behaviors was similar for male and female students, and formal tests for an interaction (Table 2) were not statistically significant.

Male participants who attended a high-performing public charter high school reported better physical health and lower BMI (Table 3). Their female counterparts had worse physical health outcomes than those who attended lower-performing high schools. Among male participants across all follow-up survey waves, the estimated proportion who reported being in fair or poor health (compared with good, very good, or excellent health) was 13.33% in the intervention group vs 23.01% in the control group (longitudinal TOT model difference −9.67% [95% CI, −18.30% to −1.05%; *P *= .03]), equaling a 42.05% lower rate. However, among female participants, rates of fair or poor health in the intervention compared with the control group were 25.43% vs 15.51% at age 20 (TOT model difference, 9.92% [95% CI, −1.96% to 21.80%]; *P*=.10) and 30.29% vs 13.47% at age 21 (TOT model difference, 16.82% [95% CI, 0.36% to 33.28%]; *P* = .045). Differences in rates of obesity or overweight among male participants in the intervention group compared with the control group were −21.59% at age 20 (TOT model 95% CI, −36.10% to −7.07%; *P* = .004), −12.26% at age 21 (TOT model 95% CI, −28.67% to 4.15%; *P* = .14), and −14.38% across all years of follow up (longitudinal TOT model 95% CI, −25.74% to −3.02%; *P *= .01). In contrast, among female participants in the intervention group compared with the control group, differences in rates of obesity or overweight were 13.91% at age 20 years (TOT model 95% CI, −2.66% to 30.47%; *P* = .10), 19.30% at age 21 (TOT model 95% CI, 3.37% to 35.22%; *P* = .02), and 8.80% across all years of follow up (longitudinal TOT model 95% CI, −3.80% to 21.39%; *P* = .17) (Table 3). An interaction term between attending a high-performing public charter high school and sex was statistically significant for almost all physical health and BMI outcomes and across all statistical models.

**Table 3.** Comparison of Global Physical Health, Body Mass Index, and Obesity or Overweight at Age 20 and 21 Years in the Intervention and Control Groups^a^

Outcome per model	All participants (n = 961)				Female participants				Male participants				*P* value for sex interaction
	Control group (n = 420)	Intervention group (n = 541)	Difference (95% CI)	*P* value	Control group (n = 232)	Intervention group (n = 296)	Difference (95% CI)	*P* value	Control group (n = 188)	Intervention group (n = 245)	Difference (95% CI)	*P* value	
**Fair or poor physical health, %**													
ITT model at age 20 y	21.50	21.13	−0.37 (−5.96 to 5.22)	.90	19.77	24.53	4.76 (−2.99 to 12.51)	.23	24.50	16.24	−8.26 (−16.17 to −0.34)	.04	.01
TOT model at age 20 y	21.78	21.07	−0.72 (−10.70 to 9.27)	.89	15.51	25.43	9.92 (−1.96 to 21.80)	.10	29.46	14.92	−14.54 (−30.05 to 0.97)	.07	.07
ITT model at age 21 y	23.19	25.99	2.80 (−3.18 to 8.78)	.36	20.53	28.84	8.31 (0.11 to 16.51)	.047	27.05	22.10	−4.94 (−13.54 to 3.65)	.26	.01
TOT model at age 21 y	21.08	26.47	5.39 (−9.03 to 19.81)	.46	13.47	30.29	16.82 (0.36 to 33.28)	.045	30.08	21.35	−8.73 (−24.43 to 6.97)	.28	.05
Longitudinal TOT	17.38	18.01	0.64 (−5.96 to 7.23)	.85	12.83	21.29	8.46 (−1.49 to 18.40)	.10	23.01	13.33	−9.67 (−18.30 to −1.05)	.03	.005
**BMI percentile, %**													
ITT model at age 20 y	64.95	62.74	−2.21 (−6.36 to 1.94)	.30	64.88	67.20	2.32 (−2.78 to 7.41)	.37	65.54	56.88	−8.66 (−15.37 to −1.95)	.01	.03
TOT model at age 20 y	66.66	62.31	−4.35 (−11.82 to 3.12)	.25	62.76	67.63	4.87 (−4.32 to 14.06)	.30	70.74	55.49	−15.25 (−25.16 to −5.34)	.003	.02
ITT model at age 21 y	66.80	65.43	−1.37 (−5.43 to 2.69)	.51	66.61	68.76	2.15 (−3.00 to 7.31)	.41	67.75	60.92	−6.84 (−13.35 to −0.32)	.04	.04
TOT model at age 21 y	67.83	65.19	−2.64 (−10.01 to 4.73)	.48	64.80	69.08	4.28 (−5.79 to 14.36)	.40	71.94	59.87	−12.07 (−21.36 to −2.78)	.01	.08
Longitudinal TOT	66.68	63.12	−3.56 (−10.38 to 3.26)	.31	63.01	67.52	4.52 (−4.81 to 13.84)	.34	70.57	57.47	−13.10 (−22.94 to −3.26)	.009	.02
**Obesity or overweight, %**													
ITT model at age 20 y	43.21	42.44	−0.78 (−7.85 to 6.29)	.83	38.49	45.16	6.67 (−2.58 to 15.92)	.16	50.44	38.18	−12.26 (−22.28 to −2.24)	.02	.01
TOT model at age 20 y	44.40	42.15	−2.25 (−14.24 to 9.75)	.71	32.51	46.42	13.91 (−2.66 to 30.47)	.10	57.81	36.22	−21.59 (−36.10 to −7.07)	.004	.01
ITT model at age 21 y	44.43	47.11	2.68 (−4.21 to 9.58)	.45	41.01	50.54	9.53 (0.29 to 18.77)	.04	49.37	42.42	−6.94 (−17.14 to 3.26)	.18	.03
TOT model at age 21 y	42.43	47.56	5.13 (−7.78 to 18.04)	.44	32.91	52.20	19.30 (3.37 to 35.22)	.02	53.62	41.36	−12.26 (−28.67 to 4.15)	.14	.03
Longitudinal TOT	35.57	33.71	−1.86 (−10.34 to 6.63)	.67	28.17	36.97	8.80 (−3.80 to 21.39)	.17	43.67	29.28	−14.38 (−25.74 to −3.02)	.01	.009

Abbreviations: BMI, body mass index; ITT, intent-to-treat; TOT, treatment on the treated.

^a^ The ITT analyses were based on mixed-effects models, with the intervention and control group assignments based on the admissions lottery. The TOT models used instrumental variables analysis, with the admissions lottery assignment as the instrument variable and attendance in a high-performing school (defined as school-level test scores in the top tertile of public high schools in Los Angeles County) as the exposure. Longitudinal models used all outcome measures for up to 5 waves of follow-up surveys, adjusted for clustering at the participant level. All models were adjusted for sex, Latino ethnicity, US birth, native English language speaker, grade 8 grade point average, risk set based on which high schools the student applied to, parental birthplace, 1 or more parental full-time employment status, parenting style at home, and family structure.

Among male and female participants, the estimated proportion who reported fair or poor mental health was higher in the intervention than in the control group (20.03% vs 11.36%; TOT model longitudinal difference, 8.67% [95% CI, 0.47% to 16.88%]; *P* = .04) (Table 4). These differences appeared to be greater among female than male participants, but the interaction was not statistically significant. Few differences were observed for other mental health outcomes except for anxiety among young men. Male participants in the intervention group reported lower rates of moderate to severe anxiety level compared with male participants in the control group at age 20 years (ITT model difference, −11.23% [95% CI, −18.31% to −4.15%]; *P* = .002; TOT model difference, −13.49% [95% CI, −26.45% to −0.54%]; *P* = .04).

**Table 4.** Comparison of Mental Health Outcomes at Age 20 and 21 Years in the Intervention and Control Groups^a^

Outcome per model	All participants (n = 961)				Female participants				Male participants				*P* value for sex interaction
	Control group (n = 420)	Intervention group (n = 541)	Difference (95% CI)	*P* value	Control group (n = 232)	Intervention group (n = 296)	Difference (95% CI)	*P* value	Control group (n = 188)	Intervention group (n = 245)	Difference (95% CI)	*P* value	
**Fair or poor mental health, %**													
ITT model at age 20 y	12.11	17.65	5.55 (0.08 to 11.01)	.05	15.05	20.20	5.15 (−2.59 to 12.89)	.19	9.51	13.45	3.93 (−2.51 to 10.37)	.23	.60
TOT model at age 20 y	8.21	17.73	9.52 (0.29 to 18.75)	.04	11.08	19.78	8.70 (−6.84 to 24.24)	.27	7.15	14.08	6.92 (−4.38 to 18.22)	.23	.89
ITT model at age 21 y	15.66	19.82	4.16 (−1.13 to 9.45)	.12	16.22	23.77	7.55 (0.05 to 15.06)	.05	15.41	14.71	−0.70 (−8.06 to 6.66)	.85	.20
TOT model at age 21 y	12.52	20.54	8.01 (−0.25 to 16.28)	.06	9.80	25.09	15.29 (0.86 to 29.72)	.04	15.84	14.60	−1.24 (−12.51 to 10.03)	.83	.23
Longitudinal TOT	11.36	20.03	8.67 (0.47 to 16.88)	.04	11.40	23.28	11.89 (−1.02 to 24.80)	.07	12.43	15.36	2.94 (−6.73 to 12.60)	.55	.29
**Depression score, %**													
ITT model at age 20 y	23.61	20.77	−2.84 (−8.47 to 2.79)	.32	25.23	24.69	−0.54 (−8.55 to 7.46)	.89	22.45	15.37	−7.09 (−14.91 to 0.74)	.08	.62
TOT model at age 20 y	25.76	20.26	−5.49 (−17.44 to 6.46)	.37	25.72	24.59	−1.14 (−18.81 to 16.54)	.90	26.71	14.24	−12.48 (−28.45 to 3.50)	.13	.46
ITT age 21	24.41	25.97	1.56 (−4.36 to 7.49)	.60	27.49	31.83	4.34 (−4.11 to 12.80)	.31	18.60	17.72	−0.89 (−9.88 to 8.10)	.85	.34
TOT age 21	23.22	26.24	3.02 (−6.72 to 12.75)	.54	23.80	32.59	8.79 (−3.22 to 20.80)	.15	23.41	17.75	−5.66 (−15.42 to 4.11)	.26	.13
Longitudinal TOT	21.74	20.87	−0.87 (−7.31 to 5.58)	.79	25.51	25.28	−0.24 (−10.72 to 10.25)	.96	17.69	15.42	−2.28 (−9.54 to 4.99)	.54	.79
**Moderate to severe anxiety score, %**													
ITT model at age 20 y	12.48	9.98	−2.50 (−6.82 to 1.81)	.26	11.31	12.88	1.57 (−4.61 to 7.74)	.62	17.21	5.98	−11.23 (−18.31 to −4.15)	.002	.20
TOT model at age 20 y	14.37	9.53	−4.84 (−12.35 to 2.67)	.21	9.91	13.17	3.27 (−5.97 to 12.50)	.49	18.60	5.11	−13.49 (−26.45 to −0.54)	.04	.20
ITT model at age 21 y	13.24	15.97	2.72 (−2.17 to 7.61)	.28	14.53	17.32	2.79 (−4.03 to 9.61)	.42	12.42	13.19	0.76 (−6.82 to 8.34)	.84	.67
TOT model at age 21 y	11.19	16.44	5.25 (−3.27 to 13.76)	.23	12.16	17.81	5.64 (−4.15 to 15.44)	.26	12.25	13.62	1.37 (−10.96 to 13.70)	.83	.67
Longitudinal TOT	14.02	14.05	0.02 (−7.12 to 7.17)	.99	12.34	16.60	4.27 (−6.11 to 14.65)	.42	16.45	10.41	−6.04 (−16.01 to 3.93)	.24	.20
**Self-efficacy, %**													
ITT model at age 20 y	33.29	33.36	0.07 (−0.52 to 0.66)	.81	33.36	32.97	−0.39 (−1.16 to 0.38)	.32	33.23	33.83	0.61 (−0.31 to 1.52)	.19	.10
TOT model at age 20 y	33.24	33.38	0.14 (−1.01 to 1.29)	.81	33.71	32.89	−0.82 (−2.42 to 0.79)	.32	32.86	33.93	1.07 (−0.31 to 2.44)	.13	.22
ITT model at age 21 y	33.75	33.58	−0.18 (−0.74 to 0.39)	.54	33.79	33.20	−0.59 (−1.33 to 0.14)	.11	33.68	34.05	0.37 (−0.50 to 1.24)	.40	.10
TOT model at age 21 y	33.89	33.55	−0.34 (−1.37 to 0.68)	.51	34.30	33.10	−1.20 (−2.41 to 0.01)	.05	33.45	34.11	0.65 (−0.81 to 2.12)	.38	.15
Longitudinal TOT	33.30	32.98	−0.32 (−1.06 to 0.41)	.39	33.66	32.74	−0.92 (−1.97 to 0.13)	.09	33.04	33.22	0.18 (−0.82 to 1.18)	.73	.14
**Hopelessness,%**													
ITT model at age 20 y	10.22	10.42	0.20 (−0.32 to 0.71)	.46	10.30	10.74	0.44 (−0.25 to 1.13)	.21	10.21	10.14	−0.06 (−0.80 to 0.67)	.86	.39
TOT model at age 20 y	10.14	10.48	0.33 (−0.77 to 1.44)	.56	9.96	10.79	0.83 (−0.89 to 2.56)	.35	10.24	10.13	−0.11 (−1.26 to 1.03)	.85	.52
ITT model at age 21 y	10.27	10.78	0.51 (0.00 to 1.02)	.05	10.38	10.99	0.61 (−0.06 to 1.29)	.08	10.13	10.53	0.39 (−0.38 to 1.17)	.32	.45
TOT model at age 21 y	9.88	10.87	0.99 (−0.13 to 2.11)	.08	9.86	11.10	1.24 (0.06 to 2.43)	.04	9.89	10.59	0.69 (−0.82 to 2.21)	.37	.65
Longitudinal TOT	8.14	8.52	0.38 (−0.16 to 0.92)	.17	8.09	8.52	0.43 (−0.39 to 1.24)	.30	8.23	8.50	0.27 (−0.44 to 0.98)	.46	.42

Abbreviations: ITT, intent-to-treat; TOT, treatment on the treated.

^a^ The ITT analyses were based on mixed-effects models, with the intervention and control group assignments based on the admissions lottery. The TOT models used instrumental variables analysis, with the admissions lottery assignment as the instrument variable and attendance in a high-performing school (defined as school-level test scores in the top tertile of public high schools in Los Angeles County) as the exposure. Longitudinal models used all outcome measures for up to 5 waves of follow-up surveys, adjusted for clustering at the participant level. All models were adjusted for sex, Latino ethnicity, US birth, native English language speaker, grade 8 grade point average, risk set based on which high schools the student applied to, parental birthplace, 1 or more parental full-time employment status, parenting style at home, and family structure.

We hypothesized that high school outcomes might mediate the association between attending a high-performing public charter high school and outcomes in young adulthood. However, additional analyses controlling for high school completion, grade 11 standardized test scores, high school GPA, and matriculation to a 4-year college did not significantly change the results (eTables 1, 2, and 3 in the Supplement).

## Discussion

To date, few interventions targeting the social determinants of health have been tested. In the present cohort study, we used the random admissions lottery system at several high-performing public charter high schools in Los Angeles to examine the health outcomes of 2 comparable cohorts of students exposed to different academic environments. We found that attending a high-performing high school was associated with substantial benefits across several health and behavioral outcomes. Specifically, we observed lower rates of hazardous or dependent AUD, cannabis misuse, and delinquent behaviors. We also observed better self-reported physical health and lower BMI among male participants who attended high-performing public charter high schools.

These findings are important given the magnitude of the differences observed. For example, we estimated that among all participants, the intervention group had 53.33% lower rates of risky alcohol misuse and 53.25% lower rates of delinquent behaviors at age 21 years, compared with control participants. Among male participants, rates of reporting fair or poor physical health was 42.05% lower than in control participants. Even a conservative estimate from the ITT analysis indicated a 33.71% reduction in fair or poor physical health by young adulthood. We estimated that, among male participants, attending a high-performing public charter high school was associated with reduced rates of obesity or overweight by 32.94% over the course of the study follow-up (29.28% vs 43.67%). Furthermore, these outcome measures, including problem drinking, self-reported health, and obesity, are important and widely used factors in all-cause mortality.^30,31,32,33^

Despite improvement in physical health and BMI among young men, rates of obesity or overweight and fair or poor physical health were worse among young women in the intervention group vs control group. The differential effect of educational interventions with better health in boys has been observed in the Perry Preschool Project and Carolina Abecedarian studies,^5,34,35^ but reasons for this finding are unclear. One explanation is that higher-performing schools raise expectations for success, potentially creating greater tension around decisions about education, career, and family. These expectations may differ for female and male participants in the present study. They may also cope differently with these expectations, possibly leading young women to experience more stress and worse physical health.

The precise mechanism of how the higher-performing high schools in this study were associated with better health outcomes in male participants is unknown, but the participating charter schools shared several characteristics. All of these schools were relatively small, with fewer than 150 students per grade. They had a structure that was based on minimizing total student load, defined as the total number of students a particular teacher is responsible for in a single semester.^36^ Thus, a teacher in a charter school who is in charge of 5 periods with 30 students each has a total student load of 150 compared with a teacher in a traditional public school who has a much higher total student load. Lower total student load may help teachers monitor and support their students, which is associated with better academic and behavioral outcomes.^37,38,39^ In addition, the participating schools were public charter schools and had more local autonomy over staffing and curriculum, and none of these schools had special health promotion programs. Although it may be argued that charter schools attract more engaged, higher-performing students, existing evidence suggests otherwise.^40^

To understand the association of attendance at high-performing public charter high schools with substance use disorder and health outcomes, we conducted analyses that controlled for intermediate educational outcomes. However, we found that the outcomes were not mediated by better academic achievement. Other possible mechanisms are greater support from teachers and other adults, more structured school environments, higher academic expectations and long-term goal setting, and less exposure to peers who engage in risky behaviors.

Finding effective, affordable, and scalable solutions to combating poverty and its adverse implications for health is enormously challenging. Schools may be part of the solution, as they are well-established social institutions, and education is broadly accepted as a fundamental right for all children. Although views may differ about school funding and best practices, school is an everyday part of almost every child’s life, and it starts fortuitously at an early life stage when its long-term role in health trajectories can be shaped.

### Strengths and Limitations

This study has several strengths. First, it examined 5 different high-performing public charter high schools serving a wide geographic area of low-income neighborhoods in Los Angeles. Participants previously attended 147 different high schools, which represents a variety of school environments. Second, the study’s natural experimental design avoids the selection bias inherent in observational studies. The ITT and TOT models also produced qualitatively similar results. The ITT estimates were more conservative and may underestimate the true outcome because of the crossover between the intervention and control groups. In contrast to an as-treated analysis, which ignores the random lottery assignment and is subject to selection bias, the TOT model uses instrumental variables both to account for the random lottery and to adjust for crossovers to better approximate a treatment outcome without bias from crossover or selection.^27,28,29^ Third, some previous natural experiments of charter schools examined the association of these schools with health outcomes, but these studies focused on only 1 charter school.^29,41^ These studies also used a cross-sectional design, sampling students several years after high school graduation, which may have increased recruitment bias. In contrast, the present study prospectively sampled students at the time of the lottery and followed the cohort over several years.

This study also has several limitations. First, it relied on self-reported data. Furthermore, because participants were not blinded to the study intervention, social desirability bias may have occurred. However, there was no evidence of systematically different responses by study group or sex, and self-reported height and weight in this age group have been found to be accurate in other studies.^42,43^ Second, despite the high yearly retention rates (>95%), control participants were slightly less likely than those in the intervention group to be retained. Those lost to attrition were more likely to be male participants and to have worse baseline academic performance, indicating higher risk. Thus, retention bias would likely lead to underestimated rates of poor health or health behaviors in the sample overall and bias estimates toward the null for analyses in which the intervention group appeared to do better than the control group. Although the random lottery assignment likely reduced the potential for bias in the study groups, unobserved socioeconomic and other factors could have confounded the results through differential attrition. Third, we studied public charter high schools because an admissions lottery system is required by California state law. However, many other types of successful school models exist, and not all public charter schools perform better than traditional public schools. Thus, the results may not generalize to other school models or suggest that all charter schools have the same health outcome. The findings may not generalize to students from higher-income families. Fourth, we identified high-performing public charter high schools using a composite measure of standardized test scores, attendance, and graduation rate, but better school performance metrics likely exist.

## Conclusion

The results of this study suggest that attending high-performing public charter high schools is associated with improvements in a range of health behaviors and health outcomes, which is particularly impressive given that the marginal cost of this intervention may be zero.^44^ Thus, costs are not necessarily a barrier with this intervention. Furthermore, high-performing public charter schools may play a role in reducing the rates of substance use disorder and, for male students, reducing incidence of obesity or overweight, 2 substantial and intransigent public health problems. It is concerning that worse physical health outcomes were observed among young women attending high-performing public charter high schools, and further inquiry into this finding is imperative. Ultimately, improving schools is a potentially effective and scalable strategy to improve health.

## Notes

Supplement. **eTable 1.** Comparison of Control and Intervention Groups in Alcohol and Cannabis Use and Delinquent Behaviors at Age 20 and 21, Adjusted for Demographics, Family Characteristics and High School Educational Outcomes **eTable 2.** Comparison of Control and Intervention Groups in Global Physical Health, Body Mass Index and Obesity at Age 20 and 21, Adjusted for Demographics, Family Characteristics and High School Educational Outcomes **eTable 3.** Comparison of Control and Intervention Groups in Mental Health Outcomes at Age 20 and 21, Adjusted for Demographics, Family Characteristics and High School Educational Outcomes
