# Errors in the Figure and Table 4

**DOI:** 10.1001/jamanetworkopen.2022.42503

**Published:** 2022-10-31

In the Original Investigation titled “Association of Attending a High-Performing High School With Substance Use Disorder Rate and Health Outcomes in Young Adults,”^1^ published on October 6, 2022, there were errors in the numbers of participants in the control and intervention groups at grade 12 listed in the Figure. In addition, the self-efficacy and hopelessness outcomes shown in Table 4 are scores rather than percentages. This article has been corrected.^1^
