# Supplementary material for: Association of Attending a High-Performing High School With Substance Use Disorder Rate and Health Outcomes in Young Adults
Source: JAMA Netw Open. 2022 Oct 6;5(10):e2235083. doi: 10.1001/jamanetworkopen.2022.35083 (PMC9539718; doi:10.1001/jamanetworkopen.2022.35083)
Supplement: Supplement. — eTable 1. Comparison of Control and Intervention Groups in Alcohol and Cannabis Use and Delinquent Behaviors at Age 20 and 21, Adjusted for Demographics, Family Characteristics and High School Educational Outcomes eTable 2. Comparison of Control and Intervention Groups in Global Physical Health, Body Mass Index and Obesity at Age 20 and 21, Adjusted for Demographics, Family Characteristics and High School Educational Outcomes eTable 3. Comparison of Control and Intervention Groups in Mental Health Outcomes at Age 20 and 21, Adjusted for Demographics, Family Characteristics and High School Educational Outcomes [file jamanetwopen-e2235083-s001.pdf]

Supplemental Online Content

Wong MD, Meza BPL, Dosanjh KK, et al. Association of attending a high-performing high school with substance use disorder rate and health outcomes in young adults. *JAMA Netw Open*. 2022;5(10):e2235083. doi:10.1001/jamanetworkopen.2022.35083

eTable 1. Comparison of Control and Intervention Groups in Alcohol and Cannabis Use and Delinquent Behaviors at Age 20 and 21, Adjusted for Demographics, Family Characteristics and High School Educational Outcomes

eTable 2. Comparison of Control and Intervention Groups in Global Physical Health, Body Mass Index and Obesity at Age 20 and 21, Adjusted for Demographics, Family Characteristics and High School Educational Outcomes

eTable 3. Comparison of Control and Intervention Groups in Mental Health Outcomes at Age 20 and 21, Adjusted for Demographics, Family Characteristics and High School Educational Outcomes

This supplemental material has been provided by the authors to give readers additional information about their work.

© 2022 Wong MD et al. *JAMA Network Open.*

eTable 1. Comparison of Control and Intervention Groups in Alcohol and Cannabis Use and Delinquent Behaviors at Age 20 and 21, Adjusted for Demographics, Family Characteristics and High School Educational Outcomes

	All			Females			Males		
**Outcome/Model**	**Control**	**Intervention**	**Difference (95%CI)**	**Control**	**Intervention**	**Difference (95%CI)**	**Control**	**Intervention**	**Difference (95%CI)**
**Alcohol use-Hazardous/Dependent (%)**									
ITT age 20	**9.15**	**5.45**	**-3.70 (-7.26, -0.14)****	9.33	5.76	-3.57 (-8.52, 1.37)	8.74	5.23	-3.51 (-8.65, 1.63)
TOT age 20	**12.02**	**4.76**	**-7.26 (-13.39, -1.14)****	12.73	5.00	-7.73 (-17.84, 2.39)	10.86	4.66	-6.20 (-15.03, 2.63)
ITT age 21	9.41	5.66	-3.75 (-7.65, 0.15)*	9.06	7.13	-1.92 (-7.57, 3.72)	9.52	6.58	-2.94 (-8.48, 2.60)
TOT age 21	**12.78**	**4.89**	**-7.89 (-15.18, -0.59)****	**13.71**	**4.09**	**-9.61 (-18.72, -0.51)****	11.34	6.12	-5.22 (-15.22, 4.78)
TOT longitudinal	**12.52**	**4.97**	**-7.55 (-13.58, -1.52)****	13.02	4.50	-8.51 (-17.44, 0.41)*	11.64	5.78	-5.85 (-13.91, 2.20)
**Cannabis misuse score**									
ITT age 20	**13.62**	**9.98**	**-3.64 (-6.13, -1.15)*****	11.48	10.10	-1.39 (-4.83, 2.05)	**15.72**	**11.08**	**-4.65 (-8.46, -0.83)****
TOT age 20	**16.44**	**9.30**	**-7.14 (-12.23, -2.04)*****	14.12	8.64	-5.48 (-12.84, 1.88)	**18.53**	**10.32**	**-8.21 (-13.87, -2.55)*****
ITT age 21	11.38	10.36	-1.03 (-3.55, 1.49)	10.30	9.54	-0.75 (-3.89, 2.38)	12.35	10.98	-1.37 (-5.43, 2.68)
TOT age 21	12.30	10.10	-2.20 (-5.78, 1.39)	10.96	9.40	-1.56 (-6.79, 3.66)	13.41	11.18	-2.23 (-7.01, 2.54)
TOT longitudinal	**9.16**	**5.68**	**-3.47 (-6.62, -0.32)****	8.23	5.38	-2.85 (-7.21, 1.52)	9.71	6.32	-3.39 (-7.71, 0.92)
**Delinquent behaviors (%)**									
ITT age 20	**13.42**	**8.27**	**-5.14 (-9.64, -0.65)****	12.39	7.06	-5.33 (-10.88, 0.21)*	13.41	9.99	-3.42 (-10.19, 3.34)
TOT age 20	**17.93**	**6.98**	**-10.95 (-19.94, -1.95)****	**17.46**	**5.93**	**-11.53 (-22.21, -0.85)****	17.79	8.66	-9.14 (-21.12, 2.84)
ITT age 21	9.11	5.62	-3.49 (-7.11, 0.13)*	7.06	4.94	-2.12 (-6.66, 2.42)	10.92	7.13	-3.80 (-9.52, 1.93)
TOT age 21	**11.83**	**5.03**	**-6.80 (-13.09, -0.51)****	8.94	4.54	-4.40 (-13.81, 5.01)	13.28	6.53	-6.74 (-16.36, 2.87)
TOT longitudinal	10.86	10.97	0.11 (-4.87, 5.10)	10.34	9.26	-1.08 (-7.85, 5.69)	11.11	13.28	2.16 (-4.82, 9.15)

eTable 1 Legend.
Results in bold indicate p≤0.05.
*p<0.10
** p≤0.05
** p≤0.001
Intent-to-treat (ITT) analyses are based on mixed effects models with intervention and control group assignment based on the admissions lottery. Treatment on the Treated (TOT) models employ instrumental variables analysis using the admissions lottery assignment as the instrument and attendance in a high-performing school (defined as school-level test scores in the top tertile of public high schools in Los Angeles County) as the exposure. Longitudinal models use all outcomes measures across up to 5 waves of follow-up surveys, adjusted for clustering at the subject level. All models adjust for sex, Latino ethnicity, U.S. birth, native English language, 8th grade grade point average, risk set based on which high schools the student applied to,

© 2022 Wong MD et al. *JAMA Network Open.*

parental birthplace, 1 or more parent working full time, parenting style at home, family structure, high school GPA, receipt of a high school diploma, 11th grade English and math standardized test scores, and matriculation to a 4-year university.

© 2022 Wong MD et al. *JAMA Network Open.*

eTable 2. Comparison of Control and Intervention Groups in Global Physical Health, Body Mass Index and Obesity at Age 20 and 21, Adjusted for Demographics, Family Characteristics and High School Educational Outcomes

	All			Females			Males		
**Model/Outcome**	**Control**	**Intervention**	**Difference (95%CI)**	**Control**	**Intervention**	**Difference (95%CI)**	**Control**	**Intervention**	**Difference (95%CI)**
**Physical health-fair/poor (%)**									
ITT age 20	22.14	20.65	-1.49 (-7.15, 4.17)	20.93	23.65	2.72 (-5.18, 10.62)	**24.84**	**15.99**	**-8.85 (-16.82, -0.88)****
TOT age 20	23.30	20.37	-2.93 (-13.07, 7.22)	18.34	24.23	5.89 (-7.09, 18.87)	30.19	14.56	-15.62 (-31.93, 0.68)*
ITT age 21	23.73	25.58	1.85 (-4.19, 7.89)	21.34	28.29	6.95 (-1.45, 15.35)	26.90	22.21	-4.69 (-13.28, 3.89)
TOT age 21	22.32	25.90	3.58 (-11.62, 18.78)	15.20	29.53	14.34 (-5.27, 33.94)	29.81	21.48	-8.33 (-24.12, 7.46)
TOT longitudinal	18.09	17.69	-0.40 (-7.16, 6.35)	14.12	20.72	6.60 (-3.71, 16.91)	**22.91**	**13.43**	**-9.48 (-18.17, -0.79)****
**Body mass index (kg/m2)**									
ITT age 20	64.98	62.69	-2.29 (-6.45, 1.87)	64.72	67.28	2.56 (-2.65, 7.77)	**65.27**	**57.12**	**-8.15 (-14.84, -1.45)****
TOT age 20	66.75	62.26	-4.49 (-12.16, 3.18)	62.21	67.83	5.62 (-4.09, 15.32)	**70.19**	**55.80**	**-14.39 (-24.62, -4.15)*****
ITT age 21	66.51	65.61	-0.89 (-5.00, 3.21)	66.10	69.07	2.97 (-2.25, 8.18)	67.32	61.25	-6.07 (-12.55, 0.42)*
TOT age 21	67.20	65.45	-1.75 (-10.22, 6.72)	63.45	69.61	6.16 (-5.06, 17.38)	**71.08**	**60.30**	**-10.77 (-20.95, -0.59)****
TOT longitudinal	66.20	63.36	-2.84 (-9.83, 4.16)	62.44	67.78	5.35 (-4.50, 15.20)	**69.37**	**58.11**	**-11.26 (-21.25, -1.27)****
**Overweight/obese (%)**									
ITT age 20	43.31	42.51	-0.80 (-7.65, 6.06)	37.92	45.58	7.66 (-1.81, 17.13)	**49.70**	**38.75**	**-10.95 (-20.93, -0.96)****
TOT age 20	43.93	42.36	-1.57 (-13.19, 10.05)	30.63	47.20	16.56 (-0.85, 33.98)*	**56.31**	**36.97**	**-19.34 (-33.66, -5.02)*****
ITT age 21	43.62	47.71	4.09 (-2.84, 11.01)	**39.58**	**51.63**	**12.05 (2.61, 21.49)****	48.40	43.14	-5.26 (-15.36, 4.84)
TOT age 21	40.46	48.46	8.00 (-5.22, 21.21)	**28.91**	**53.91**	**25.01 (8.01, 42.00)*****	51.66	42.32	-9.34 (-25.91, 7.23)
TOT longitudinal	34.69	34.14	-0.55 (-9.21, 8.12)	26.84	37.58	10.74 (-2.63, 24.10)	**42.00**	**30.17**	**-11.83 (-23.30, -0.37)****

eTable 2 Legend

Results in bold indicate p≤0.05.
*p<0.10
** p≤0.05
** p≤0.001
Intent-to-treat (ITT) analyses are based on mixed effects models with intervention and control group assignment based on the admissions lottery. Treatment on the Treated (TOT) models employ instrumental variables analysis using the admissions lottery assignment as the instrument and attendance in a high-performing school (defined as school-level test scores in the top tertile of public high schools in Los Angeles County) as the exposure. Longitudinal models use all outcomes measures across up to 5 waves of follow-up surveys, adjusted for clustering at the subject level. All models adjust for sex, Latino ethnicity, U.S. birth, native English language, 8th grade grade point average, risk set based on which high schools the student applied to, parental birthplace, 1 or more parent working full time, parenting style at home, family structure, high school GPA, receipt of a high school diploma, 11th grade English and math standardized test scores, and matriculation to a 4-year university.

© 2022 Wong MD et al. *JAMA Network Open.*

eTable 3. Comparison of Control and Intervention Groups in Mental Health Outcomes at Age 20 and 21, Adjusted for Demographics, Family Characteristics and High School Educational Outcomes

	All			Females			Males		
**Outcome/Model**	**Control**	**Intervention**	**Difference (95%CI)**	**Control**	**Intervention**	**Difference (95%CI)**	**Control**	**Intervention**	**Difference (95%CI)**
**Mental health-fair/poor (%)**									
ITT age 20	12.68	17.72	5.04 (-0.48, 10.57)*	15.42	20.16	4.74 (-3.06, 12.54)	10.30	12.79	2.48 (-3.88, 8.84)
TOT age 20	9.69	17.01	7.32 (-2.43, 17.06)	12.08	19.33	7.25 (-9.06, 23.56)	8.80	13.19	4.38 (-7.81, 16.57)
ITT age 21	16.52	19.15	2.63 (-2.70, 7.95)	16.84	23.29	6.45 (-1.20, 14.10)*	15.94	14.30	-1.64 (-8.98, 5.69)
TOT age 21	14.49	19.63	5.13 (-2.91, 13.18)	11.13	24.51	13.39 (-0.91, 27.68)*	16.96	14.04	-2.91 (-14.23, 8.40)
TOT longitudinal	12.99	19.09	6.10 (-2.18, 14.38)	12.49	22.63	10.15 (-3.29, 23.58)	13.66	14.50	0.83 (-8.73, 10.40)
**Depression (%)**									
ITT age 20	23.45	20.91	-2.55 (-8.22, 3.13)	24.34	25.40	1.06 (-7.14, 9.25)	**22.86**	**15.05**	**-7.81 (-15.50, -0.12)****
TOT age 20	25.43	20.43	-5.00 (-16.87, 6.87)	23.34	25.62	2.29 (-13.90, 18.47)	27.58	13.79	-13.79 (-28.59, 1.00)*
ITT age 21	24.92	25.57	0.66 (-5.32, 6.64)	27.61	31.74	4.13 (-4.50, 12.76)	18.53	17.14	-1.39 (-10.46, 7.67)
TOT age 21	24.41	25.69	1.28 (-7.89, 10.45)	23.95	32.52	8.58 (-3.13, 20.28)	24.66	17.12	-7.54 (-17.27, 2.20)
TOT longitudinal	22.01	20.76	-1.24 (-7.86, 5.37)	24.99	25.52	0.53 (-10.36, 11.42)	18.29	15.13	-3.16 (-10.63, 4.31)
**Anxiety- moderate to severe (%)**									
ITT age 20	12.60	9.94	-2.66 (-7.04, 1.71)	11.22	13.05	1.83 (-4.52, 8.18)	**17.70**	**5.89**	**-11.81 (-18.91, -4.70)*****
TOT age 20	14.67	9.44	-5.23 (-13.06, 2.60)	9.47	13.44	3.96 (-5.27, 13.20)	**19.32**	**4.73**	**-14.59 (-27.74, -1.44)****
ITT age 21	13.39	15.85	2.47 (-2.48, 7.41)	14.60	17.27	2.68 (-4.34, 9.69)	12.13	12.95	0.83 (-6.96, 8.61)
TOT age 21	11.48	16.30	4.82 (-3.91, 13.55)	12.23	17.78	5.56 (-4.70, 15.82)	12.48	13.50	1.02 (-11.16, 13.20)
TOT longitudinal	14.31	13.94	-0.38 (-7.86, 7.11)	12.16	16.71	4.55 (-6.86, 15.96)	16.98	10.29	-6.69 (-16.52, 3.15)
**Self-efficacy score**									
ITT age 20	33.28	33.37	0.08 (-0.52, 0.68)	33.33	32.99	-0.35 (-1.14, 0.45)	33.28	33.79	0.51 (-0.41, 1.43)
TOT age 20	33.22	33.38	0.16 (-0.97, 1.29)	33.66	32.91	-0.75 (-2.42, 0.92)	32.97	33.87	0.90 (-0.36, 2.16)
ITT age 21	33.70	33.62	-0.09 (-0.66, 0.48)	33.73	33.26	-0.47 (-1.22, 0.28)	33.66	34.06	0.40 (-0.47, 1.26)
TOT age 21	33.77	33.60	-0.17 (-1.25, 0.90)	34.14	33.17	-0.98 (-2.26, 0.30)	33.42	34.12	0.71 (-0.74, 2.16)
TOT longitudinal	33.30	32.98	-0.32 (-1.05, 0.41)	33.58	32.77	-0.81 (-1.87, 0.26)	33.15	33.16	0.01 (-1.00, 1.02)
**Hopelessness score**									
ITT age 20	10.19	10.45	0.26 (-0.26, 0.78)	10.24	10.76	0.52 (-0.19, 1.23)	10.17	10.17	-0.00 (-0.74, 0.74)
TOT age 20	10.05	10.52	0.47 (-0.60, 1.55)	9.84	10.84	1.00 (-0.79, 2.79)	10.17	10.17	-0.00 (-1.09, 1.08)

© 2022 Wong MD et al. *JAMA Network Open.*

ITT age 21	**10.22**	**10.78**	**0.56 (0.03, 1.08)****	10.38	10.99	0.60 (-0.09, 1.30)*	10.09	10.56	0.47 (-0.30, 1.24)
TOT age 21	9.83	10.89	1.07 (-0.11, 2.24)*	9.85	11.10	1.26 (-0.04, 2.55)*	9.80	10.63	0.84 (-0.64, 2.31)
TOT longitudinal	**8.02**	**8.58**	**0.56 (0.02, 1.10)****	7.96	8.58	0.62 (-0.21, 1.45)	8.13	8.55	0.42 (-0.29, 1.13)

eTable 3 legend
Results in bold indicate p≤0.05.
*p<0.10
** p≤0.05
** p≤0.001
Intent-to-treat (ITT) analyses are based on mixed effects models with intervention and control group assignment based on the admissions lottery. Treatment on the Treated (TOT) models employ instrumental variables analysis using the admissions lottery assignment as the instrument and attendance in a high-performing school (defined as school-level test scores in the top tertile of public high schools in Los Angeles County) as the exposure. Longitudinal models use all outcomes measures across up to 5 waves of follow-up surveys, adjusted for clustering at the subject level. All models adjust for sex, Latino ethnicity, U.S. birth, native English language, 8th grade grade point average, risk set based on which high schools the student applied to, parental birthplace, 1 or more parent working full time, parenting style at home, family structure, high school GPA, receipt of a high school diploma, 11th grade English and math standardized test scores, and matriculation to a 4-year university.

© 2022 Wong MD et al. *JAMA Network Open.*
